# Glycosaminoglycan linkage region of urinary bikunin as a potentially useful biomarker for β3GalT6‐deficient spondylodysplastic Ehlers–Danlos syndrome

**DOI:** 10.1002/jmd2.12311

**Published:** 2022-06-28

**Authors:** Mahnaz Nikpour, Fredrik Noborn, Jonas Nilsson, Tim Van Damme, Olivier Kaye, Delfien Syx, Fransiska Malfait, Göran Larson

**Affiliations:** ^1^ Department of Laboratory Medicine, Sahlgrenska Academy University of Gothenburg Gothenburg Sweden; ^2^ Proteomics Core Facility, Sahlgrenska Academy University of Gothenburg Gothenburg Sweden; ^3^ Department of Biomolecular Medicine, Center for Medical Genetics Ghent University Hospital Ghent Belgium; ^4^ Centre de Rheumatologie Liège Belgium; ^5^ Laboratory of Clinical Chemistry Sahlgrenska University Hospital Gothenburg Sweden

**Keywords:** bikunin, Ehlers–Danlos syndrome, glycopeptides, glycoproteomics, glycosaminoglycan linkage region, linkeropathies, liquid chromatography, tandem mass spectrometry, β3GalT6

## Abstract

The spondylodysplastic type of Ehlers–Danlos syndrome (spEDS) is caused by genetic defects in the *B4GALT7* or *B3GALT6* genes both deranging the biosynthesis of the glycosaminoglycan linkage region of chondroitin/dermatan sulfate and heparan sulfate proteoglycans. In this study, we have analyzed the linkage regions of urinary chondroitin sulfate proteoglycans of three siblings, diagnosed with spEDS and carrying biallelic pathogenic variants of the *B3GALT6* gene. Proteoglycans were digested with trypsin, glycopeptides enriched on anion‐exchange columns, depolymerized with chondroitinase ABC, and analyzed by nLC‐MS/MS. In urine of the unaffected mother, the dominating glycopeptide of bikunin/protein AMBP appeared as only one dominating (99.9%) peak with the canonical tetrasaccharide linkage region modification. In contrast, the samples of the three affected siblings contained two different glycopeptide peaks, corresponding to the canonical tetrasaccharide and to the non‐canonical trisaccharide linkage region modifications in individual ratios of 61/38, 73/27, and 59/41. We propose that the relative distribution of glycosaminoglycan linkage regions of urinary bikunin glycopeptides may serve as a phenotypic biomarker in a diagnostic test but also as a biomarker to follow the effect of future therapies in affected individuals.

**Synopsis**

We have identified a >100‐fold relative increase in the non‐canonical trisaccharide versus the canonical tetrasaccharide linkage region of tryptic bikunin glycopeptides, prepared from urine of three siblings with identical biallelic variants of the *B3GALT6* gene and diagnosed with spondylodysplastic Ehlers–Danlos syndrome, which we propose may serve as a biomarker for this disease.

## 1 INTRODUCTION

**FIGURE 1:**
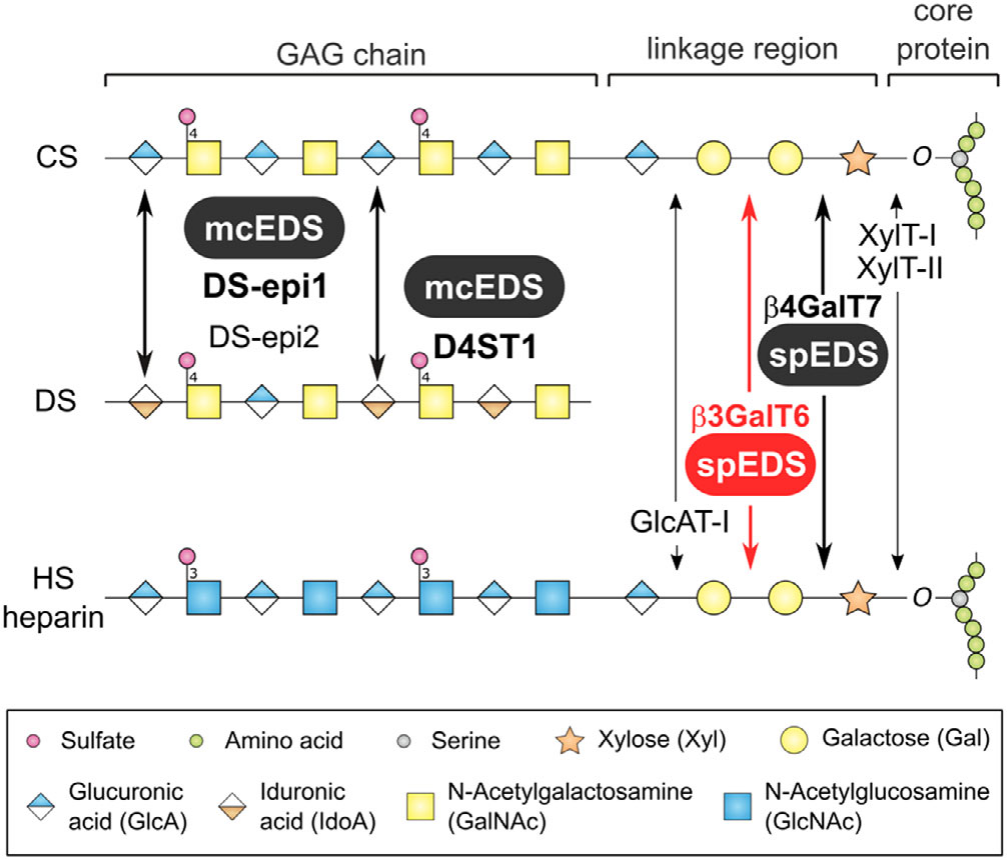
Schematic representation of proteoglycan linkage and GAG biosynthesis with the indication of the associated EDS types. The assembly of both CS/DS and HS GAGs starts with the biosynthesis of a common tetrasaccharide linkage region (GlcA‐Gal‐Gal‐Xyl‐*O*‐) at specific serine residues of the core proteins.^16^ The first step is initiated by adding a Xyl residue to a serine residue by the action of one of two xylosyltransferases XylT‐I or XylT‐II (encoded by *XYLT1* and *XYLT2*, respectively). Subsequently, two Gal and one GlcA residue are added to the growing chain by sequential actions of the enzymes β4GalT7/GalT‐I (encoded by *B4GALT7*), β3GalT6/GalT‐II (encoded by *B3GALT6*), and GlcAT‐I (encoded by *B3GAT3*)^1^. Biosynthesis of the CS/DS and HS GAG chains continues by the enzymatic addition of GlcA and GalNAc (for CS/DS) or GlcA and GlcNAc (for HS GAGs).^4^ Finally, various monosaccharides undergo a series of enzymatic modifications including epimerization, *N*‐deacetylation, *N*‐, and *O*‐sulfations to become the mature GAGs. Abbreviations: mcEDS, musculocontractural Ehlers–Danlos syndrome; spEDS, spondylodysplastic Ehlers–Danlos syndrome

The Ehlers‐Danlos syndromes (EDSs) constitute a very heterogeneous group of heritable connective tissue disorders, mainly presenting with skin hyperextensibility, joint hypermobility, and general tissue fragility.^12^ The EDS consists of 13 different types, each of which is presenting with its own characteristic phenotypes and is caused by unique disabling genetic alterations affecting anyone of 20 well‐characterized genes. The human EDS and related diseases in various model animals have recently been covered in a series of extensive reviews.^3^, ^12^, ^17^, ^19^, ^20^, ^21^ Two types of EDS are directly caused by defects in the biosynthesis of the negatively charged polysaccharides known as the glycosaminoglycans. Musculocontractural EDS (mcEDS) is caused by pathogenic variants in the *CHST14* or *DSE* genes, and spondylodysplastic EDS (spEDS) is caused by pathogenic variants in the *B4GALT7* or *B3GALT6* genes. The latter two genes are coding for two unique galactosyltransferases (β4Gal‐T7 and β3Gal‐T6, respectively) that work in sequence adding two galactose (Gal) residues to what is to become the canonical tetrasaccharide “linkage region,” common to all glycosaminoglycans of chondroitin/dermatan (CS/DS) and heparan sulfate (HS) proteoglycans, which links the glycosaminoglycan chains to the core proteins (Figure 1). Thus, due to the underlying molecular explanations, these spEDS are part of a group of syndromes that are often referred to as the “linkeropathies.”

Proteoglycans (PGs) are composed of a core protein to which one or multiple glycosaminoglycan chains (GAGs) are bound.^11^ In vertebrates and invertebrates, PGs are found in the extracellular matrix (ECM), on cell surfaces, or in intracellular granules.^8^ Proteoglycans are functionally involved in many physiological processes, such as matrix tissue support, growth factor signaling, proliferation, migration, adhesion, differentiation, and survival.^8^ Proteoglycans are categorized into chondroitin/dermatan sulfate PGs, heparin/heparan sulfate PGs, and keratan sulfate (KS) PGs according to the structures of their GAG chains.^9^ Figure 1 illustrates the biosynthesis of CS/DS and HS GAGs including the assembly of the linkage region to core proteins,^1^, ^16^ the extension of the different GAG chains, and their final modifications^4^ as well as the enzymes involved in the critical steps associated with mcEDS and spEDS pathogenicity.

The structure of the tetrasaccharide linkage region, common to CSPGs/DSPGs and HSPGs, was settled more than 50 years ago by the two groups of Roden and Lindahl et al.^10^, ^18^ and has subsequently been considered the sole structural basis for all CS/DS and HS GAGs. However, using a glycoproteomic approach we recently, and unexpectedly, identified very low amounts of a non‐canonical linkage region with a trisaccharide structure (GlcA‐Gal‐Xyl‐*O*‐) linked to Ser‐215 of bikunin (Protein AMBP, UniProtKB ID 02760) in the urine of healthy human individuals.^15^ Interestingly, we found the same trisaccharide linkage region on biglycan and on aggrecan in a *b3galt6* knock‐out zebrafish showing a typical dysplastic phenotype resembling the human spEDS‐*B3GALT6*.^2^ To find the molecular correlates to those findings in diseased humans, we hypothesized that possibly the proportion of the non‐canonical trisaccharide linkage region would be increased in patients diagnosed with spEDS. Thus, we have, using our established glycoproteomic approach,^13^, ^14^ studied the linkage regions of proteoglycans in urine samples from three siblings, diagnosed with biallelic pathogenic variants in the *B3GALT6* gene, and showed a typical spEDS phenotype.^19^ A urine sample from their unaffected mother served as a control. The nLC‐MS/MS data clearly show that the distribution of the canonical and non‐canonical linkage regions is highly deranged in the diseased siblings and that this kind of glycoproteomic analysis reveals how urinary proteoglycans, in particular bikunin, may serve as an accessible biomarker for spEDS‐*B3GALT6*, complementary to the clinical signs and genetic analyses.

## 2 MATERIALS AND METHODS

### 2.1 Definition of patients and ethical approvals

The individuals participating in this study include three previously reported siblings with biallelic pathogenic variants in the *B3GALT6* gene (NM_080605.3) and their unaffected mother (family IV in^19^). All three patients are compound heterozygous for the *B3GALT6*:c.197_253del, p.(Ala66_Arg84del) deletion variant and the *B3GALT6*:c.953C>T, p.(Pro318Leu) missense variant. Informed consent was obtained from the patients and/or their participating parents. This study was approved by the Ethics Committee of the Ghent University Hospital (Ghent, Belgium).

### 2.2 Sampling, processing, enrichment, and structural analysis of tryptic glycopeptides of urinary proteoglycans

The time of urine sampling was at ages 29 years (PIV;1), 33 years (PIV:2), 36 years (PIV:1), and 54 years (mother), respectively. Morning urine was sampled and immediately centrifuged at 2000 × *g*, for 10 min at room temperature. The supernatants were collected, frozen, and kept at −20°C until analysis. Linkage region containing glycopeptides was prepared from urinary proteoglycans using the protocol described in^14^ without any modifications. In brief, the protocol includes in‐solution trypsin digestion, enrichment by strong anion‐exchange chromatography, depolymerization with chondroitinase ABC, and a series of desalting steps before final lyophilization. This treatment typically generates glycopeptides with residual hexasaccharides of CS/DS chains, including the common linkage region attached to the original core proteins serine residues.

Glycoproteomic analyses were performed as technical triplicates on an Orbitrap Fusion Tribrid instrument and the Easy‐nLC 1200 system (Thermo Scientific). Samples were dissolved in 20 μl 3% acetonitrile and 0.2% formic acid in dH_2_0, and from this 3 μl were injected onto an Acclaim Pepmap C18 precolumn (20 × 0.1 mm I.D., 5 μm) and passed to the analytical column (350 × 0.075 mm I.D.) packed in‐house with 3 μm ReproSil‐Pur C18‐AQ particles (Dr. Maisch). The gradient was 10–50% B‐solvent (80% acetonitrile, 0.2% formic acid in dH_2_O) in A‐solvent (0.2% formic acid in dH_2_O) over 60 min at a flow rate of 300 nl/min, and then 50%–100% B over 5 min, with a final hold at 100% B for 10 min. MS scans were done in positive ionization mode at 120 000 resolution (at *m/z* 200): an automatic gain control (AGC) target value of 10^6^ and a mass range of *m/z* 600–2000. The MS/MS (MS^2^) spectra were collected in the data‐dependent mode with a duty cycle time of 3 s and a dynamic exclusion of 10 s. The most abundant 2(+) – 7(+) charged precursor ions for each MS scan were fragmented by higher‐energy collision dissociation (HCD) at normalized collision energy (NCE) levels of 20%, 30%, and 35%. The isolation window was set to 2.5 *m/z* units, the MS^2^ mass range was *m/z* 100–2000 at 30 000 resolution, and MS^2^ data were collected in profile mode.

Data analysis was performed with Mascot distiller (Matrix Science) and manual verification and complementary fragmentation analysis of the glycopeptide spectra using the Qual browser of the Xcalibur software. This glycoproteomic technique allows for identification of the type of GAG chains, their linkage regions, the identity of the original proteoglycan, and their sites for glycosylation.^13^ In this study, we also employed the ion intensities of both 2(+) and 3(+) precursor ions of relevant glycopeptides for estimation of the relative amounts of glycopeptides with structural differences found only in the linkage region, i.e., canonical versus non‐canonical linkage regions both harboring two sulfate modifications.

## 3 RESULTS

In the control urine of the mother, we identified GAG‐modified glycopeptides of 16 different CSPGs (bikunin/protein AMBP, osteopontin, neuropeptide W, CD44 antigen, laminin subunit alpha‐4, secretogranin‐1, chromogranin‐A, basement membrane‐specific heparan sulfate proteoglycan core protein, plexin domain‐containing protein 1, decorin, dermcidin, meprin A subunit alpha, bone marrow proteoglycan, HLA class II histocompatibility antigen gamma chain, membrane‐associated progesterone receptor component 1, and chondroitin sulfate proteoglycan 5; see Table S1). These glycoproteomic results are similar to what we have found previously in urine samples of healthy human individuals.^13^ Similar glycopeptides from three of those CSPGs (bikunin/protein AMBP, osteopontin, and CD44 antigen) could also be identified in urine from all three affected siblings. For bikunin/protein AMBP, the quantitatively dominating CSPG in urine,^5^ we identified GAG‐modified glycopeptides with both canonical and non‐canonical linkage regions from the three affected siblings as well as from their unaffected mother, and thus we focused our comparative study of these urinary samples on the bikunin glycopeptides.

**FIGURE 2:**
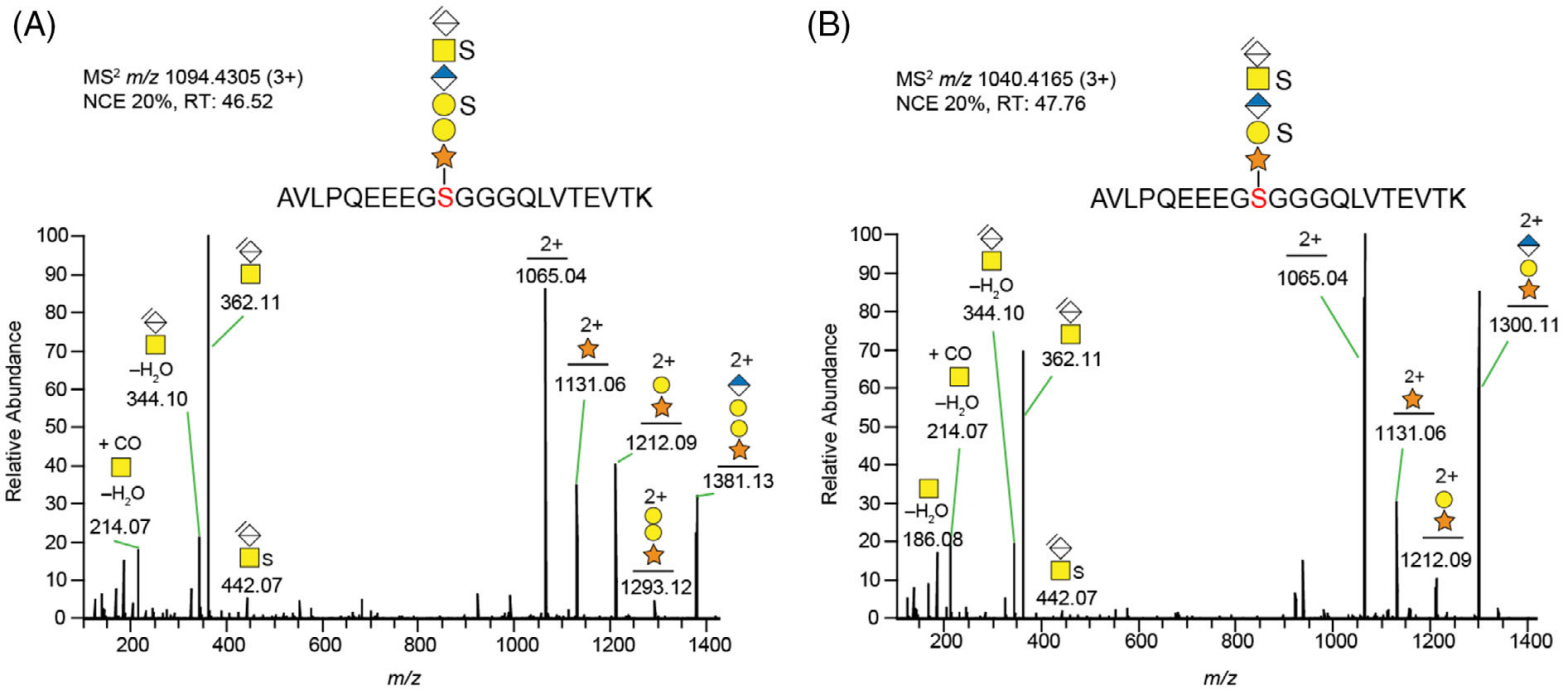
MS/MS spectra of bikunin/protein AMBP showing glycan fragmentations of two glycopeptides with either a canonical (A) or a non‐canonical (B) linkage region attached to the same peptide sequence. The urine sample analyzed was from individual PIV:1, one of three siblings diagnosed with spEDS with a β3GalT6 deficiency.^19^

The glycopeptide identified from bikunin in all samples was the tryptic glycopeptide R.AVLPQEEEGS^215^GGGQLVTEVTK.K, modified with either a disulfated hexasaccharide (6‐mer residual, with the canonical tetrasaccharide linkage region, obtained after enzymatic depolymerization with chondroitinase ABC) or a disulfated pentasaccharide (5‐mer residual with the non‐canonical trisaccharide linkage region obtained after enzymatic depolymerization; Figure 2). Other residual linkage region glycans without any sulfates or with only one sulfate modification on the same glycan of this glycopeptide, as well as other glycan modified semi‐tryptic peptides of bikunin, were additionally identified in the control sample, but not consequently in all affected sibling samples (Table S1, Figures S1–S2). Thus, only the dominating fully tryptic glycopeptides modified with the disulfated residual saccharides were used for comparisons of the relative amounts of the canonical hexasaccharide (6‐mer) and the non‐canonical pentasaccharide (5‐mer) modifications in the individual samples. As shown in Table 1, there was a dramatic increase in the relative amounts of the unique 5‐mer glycan for all three siblings, varying from 27.3% to 40.9% compared to their mother having only 0.1% of this glycan structure and 99.9% of the 6‐mer glycan structure linked to this dominating glycopeptide. For the siblings, the 6‐mer glycan was reciprocally reduced to relative amounts of 72.7%–59.1%.

**TABLE 1:** Relative distribution of canonical hexameric and non‐canonical pentameric GAG linkage regions of the dominating tryptic glycopeptide of bikunin in urine samples of three siblings diagnosed with spEDS‐B3GALT6 and their unaffected mother

	Relative distribution (%)^a^		
Individual	6‐mer	5‐mer	SD^b^
Mother	99.9	0.1	0.0%
PIV:1	61.2	38.8	6.8%
PIV:2	72.7	27.3	5.1%
PIV:3	59.1	40.9	2.7%

^a^ The calculations of relative distributions included glycopeptide precursor ions of both 2+ and 3+ charges.

^b^ The SD values were calculated from technical triplicates.

## 4 DISCUSSION

The almost complete dominance (99.9%) of the canonical hexasaccharide modification in the mother control sample fits well with what is well known for CSPGs and what we have described before for bikunin.^15^ However, the diseased sibling samples show a dramatic (>100‐fold) increase in the relative amounts of the pentasaccharide modification of the same bikunin glycopeptide. Thus, instead of adding another Gal by the β3GalT6/GalT‐II enzyme, which is deficient in these individuals, the GlcAT‐I enzyme adds on a GlcA to the inner Gal and the biosynthesis to more extended GAGs can continue. For the affected individuals, the mere presence of the hexasaccharide indicates that the block in the linkage region biosynthesis is only partial, which also can be expected from what is known from the characterization of their genetic defects, that is, a deletion and a missense variant, p.(Ala66_Arg84del) and p.(Pro318Leu), respectively, which are expected to result in only partially functional enzymes.^19^ The model of substrate competition is also supported by the fact that the non‐canonical linkage region can indeed be found in very minor amounts of bikunin also in urine of healthy individuals (reference range <0.5%). The relative amounts of these two forms of linkage regions may thus be dependent on not only the relative expression and enzymatic activity of the β3GalT6/GalT‐II enzyme but also the activity of the GlcAT‐I enzyme, as well as on other factors such as the availability of substrates and UDP‐sugars (and possibly energy and redox status of the cells producing these CSPGs). This complexity may be an explanation for the differences in relative distribution of the two forms of linkage regions seen in the three siblings, all with the same biallelic genetic deficiencies of the *B3GALT6* gene but showing somewhat different symptomatologies. The relative distribution of glycosaminoglycan linkage regions of urinary bikunin glycopeptides may thus serve not only as a phenotypic biomarker in a diagnostic test but possibly also as a biomarker to follow the effect of future therapies (e.g., enzyme replacement therapies) in affected children. In a recent publication, the Bruneel group reviewed the status of known proteoglycan inherited biosynthesis defects and the analytical tools available for diagnosis.^7^ They also summarized and commented on their own work on using Western blot, or two‐dimensional electrophoresis, for efficiently identifying different glycoforms of serum bikunin as screening methods for such diseases.^6^ However, as also commented on in their review, these methods will, in contrast to what becomes available by mass spectrometry, not provide accurate structural data on the GAG chains or on the glycopeptides of relevant proteoglycans as presented here.

Interestingly, in the urine sample of the individual PIV:1, the non‐canonical form of the linkage region also appeared on a glycopeptide of neuropeptide W (Figure S2B), without any comparable amounts of the canonical linkage region of the same peptide being found (Table S1). From this result, one may conclude that the relative distribution of these two forms of linkage regions of GAGs is not equal among all CSPGs but seems dependent on the core proteins themselves as well as on the glycosylation machinery of individual types of cells and tissues. Of note is that in the zebrafish *b3galt6* KO model, having an EDS‐like phenotype, we found the non‐canonical linkage region only on biglycan and aggrecan, whereas in the wild‐type zebrafish, the canonical linkage region of CSPGs was found not only on biglycan and aggrecan but also on epiphycan, osteopontin, and syndecan‐3 (as well as on four different HSPGs).^2^ In this model system, however, the concentration of CS/DS (as well as HS) disaccharides was significantly decreased in both bone and skin in the KO fishes compared to the wild‐type fishes, which is a likely explanation for the identification of less CSPGs and HSPGs in the mutated tissues. A similar decrease of CSPGs in the urine of the three spEDS‐B3GALT6 siblings now analyzed would also explain our identification of only variants of bikunin, the dominating CSPG in human urine, and not of all the other CSPGs found in the unaffected mother.

In conclusion, the glycoproteomic approach used here may become a suitable diagnostic method for clinical purposes but may also be helpful in deciphering the biological complexity of deranged GAG biosynthesis and the importance of linkage region modifications and core protein structures for the biosynthesis of both CSPGs and HSPGs.

## FUNDING INFORMATION

The work was supported by grants from the Swedish Research Council (2017‐00955), the Swedish state under the agreement between the Swedish government and the county councils, the ALF agreement (ALFGBG_721971) and Research Foundation Flanders (12Q5920N to Delfien Syx, 1842318N and G0A3322N to Fransiska Malfait).

## CONFLICT OF INTEREST

Mahnaz Nikpour, Fredrik Noborn, Jonas Nilsson, Tim Van Damme, Olivier Kaye, Delfien Syx, Fransiska Malfait, and Göran Larson all declare that they have no conflict of interest.

## INFORMED CONSENT

All procedures followed were in accordance with the ethical standards of the responsible committee on human experimentation (institutional and national) and with the Helsinki Declaration of 1975, as revised in 2000. Informed consent was obtained from the patients and/or parents participating in this study. The study was approved by the Ethics Committee of the Ghent University Hospital (Ghent, Belgium).

## ANIMAL RIGHTS

No animal subjects were used in this study.

## Supporting information


**Appendix S1.** Supporting Information

## Data Availability

The mass spectrometry proteomics data have been deposited to the ProteomeXchange Consortium via the PRIDE [1] partner repository with the dataset identifier PXD033230.
