# Supplementary material for: Glycosaminoglycan linkage region of urinary bikunin as a potentially useful biomarker for β3GalT6‐deficient spondylodysplastic Ehlers–Danlos syndrome
Source: JIMD Rep. 2022 Jun 28;63(5):462–7. doi: 10.1002/jmd2.12311 (PMC9458601; doi:10.1002/jmd2.12311)
Supplement: Supplementary file 1 — Appendix S1. Supporting Information [file JMD2-63-462-s001.docx]

**Supplementary material**

**Table S1.** Summary of glycopeptides of CSPGs identified in the urine samples of a healthy mother and her three siblings diagnosed with spEDS-*B3GALT6*.

| **Protein name** | **UniProt ID** | **Glycan Modification^a^ mother** | **Glycan**  **Modification PIV:1** | **Glycan**  **Modification PIV:2** | **Glycan Modification PIV:3** |
| --- | --- | --- | --- | --- | --- |
| **Protein AMBP** | P02760 | 6- and 5-mer | 6- and 5-mer | 6- and 5-mer | 6- and 5-mer |
| **Osteopontin** | P10451 | 6-mer | 6-mer | 6-mer | 6-mer |
| Neuropeptide W | Q8N729 | 6-mer | 5-mer |  | 6-mer |
| Laminin subunit alpha-4 | Q16363 | 6-mer | 6-mer |  |  |
| **CD44 antigen** | P16070 | 6-mer | 6-mer | 6-mer | 6-mer |
| Secretogranin-1 | P05060 | 6-mer |  | 6-mer |  |
| Chromogranin-A | P10645 | 6-mer |  |  |  |
| Basement membrane-specific heparan sulfate proteoglycan core protein | P98160 | 6-mer | 6-mer |  |  |
| Plexin domain-containing protein 1 | Q8IUK5 | 6-mer |  |  |  |
| Decorin | P07585 | 6-mer | 6-mer |  |  |
| Dermcidin | P81605 | 6-mer |  |  |  |
| Meprin A subunit alpha | Q16819 | 6-mer |  |  |  |
| Bone marrow proteoglycan | P13727 | 6-mer |  |  |  |
| HLA class II histocompatibility antigen gamma chain | P04233 | 6-mer |  |  |  |
| Membrane-associated progesterone receptor component 1 | O00264 | 6-mer |  |  |  |
| Chondroitin sulfate proteoglycan 5 | O95196 |  | 6-mer |  |  |

^a^ The residual CS modifications (after chondroitinase ABC depolymerizations) are described as hexasaccharides (6-mers with the canonical tetrasaccharide linkage region) and pentasaccharides (5-mers with the non-canonical trisaccharide linkage region). Proteins marked in bold were identified from tryptic glycopeptides found in all four samples.


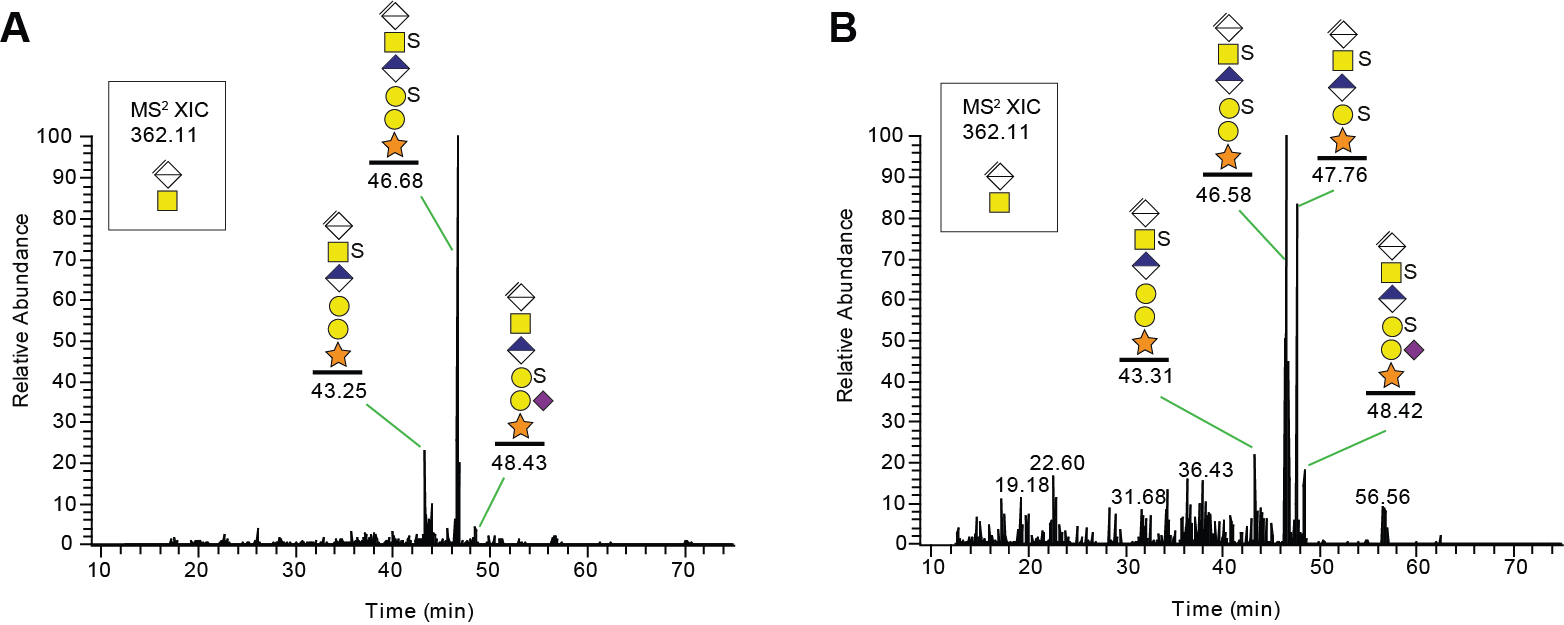


**Figure S1**. Extracted ion chromatograms of the diagnostic oxonium ion at *m/z* 362 (ΔHexA-HexNAc) showing the dominant glycoforms linked to the tryptic peptide AVLPQEEEGS^215^GGGQLVTEVTK of bikunin/protein AMBP from urine samples of **A**) the healthy mother and **B**) one of her children, PIV:1, diagnosed with spEDS-*B3GALT6*. The glycopeptides eluting at 46-48 min and corresponding to the hexameric and pentameric glycan residuals, obtained after enzymatic treatment with trypsin and chondroitinase ABC, respectively, were used for calculating the difference in relative abundances of these glycoforms (Table 1).

**
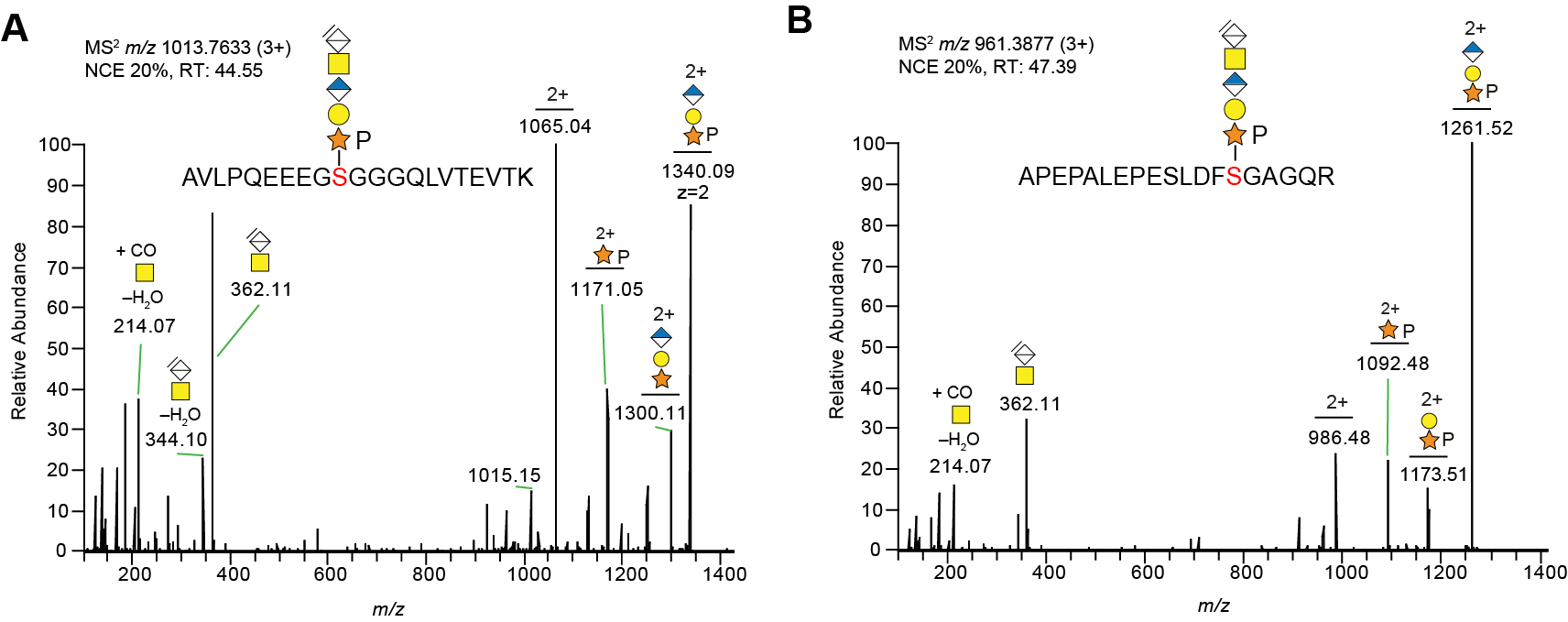
**

**Figure S2.** MS/MS spectra of two minor glycopeptides both carrying a phosphorylated Xyl residue linked to **A)** S^215^ of the peptide AVLPQEEEGS^215^GGGQLVTEVTK of bikunin and **B)** to one of the two Ser residues of the peptide APEPALEPESLDFS^133^GAGQR of neuropeptide W. The exact glycosylation site on this peptide was not possible to deduce from this analysis, but we have shown from samples from human cerebrospinal fluid that neuropeptide W may be glycosylated at S^133^ (Noborn et al. 2015). The two spectra were taken from the same analytical run as in Figures 1 and S1, i.e., from urine of individual PIV:1, diagnosed with spEDS-*B3GALT6*. The collision energy was set to a NCE level of 20% to enable optimal glycan fragmentation and identification. Amino acid sequence was settled from spectra recorded at higher NCE levels, i.e., 35% (not shown). The fragment ions in panel A) at *m/z* 1171.05 and *m/z* 1340.09 and in panel B) at *m/z* 1092.48, 1173.51 and *m/z* 1261.52 are diagnostic for these particular peptides carrying a phosphorylated Xyl in the trisaccharide linkage region.
